# Association between plasma levels of hyaluronic acid and functional outcome in acute stroke patients

**DOI:** 10.1186/1742-2094-11-101

**Published:** 2014-06-10

**Authors:** Sung-Chun Tang, Shin-Joe Yeh, Li-Kai Tsai, Chaur-Jong Hu, Li-Ming Lien, Giia-Sheun Peng, Wei-Shiung Yang, Hung-Yi Chiou, Jiann-Shing Jeng

**Affiliations:** 1 Stroke Center and Department of Neurology, National Taiwan University Hospital, Taipei, Taiwan; 2 Department of Neurology, Taipei Medical University Hospital and Shuang Ho Hospital, Taipei, Taiwan; 3 Department of Neurology, Shin Kong Wu Ho-Su Memorial Hospital, Taipei, Taiwan; 4 Department of Neurology, Tri-Service General Hospital, Taipei, Taiwan; 5 Internal Medicine, National Taiwan University Hospital, Taipei, Taiwan; 6 Graduate Institute of Clinical Medicine, College of Medicine, National Taiwan University, Taipei, Taiwan; 7 School of Public Health, Taipei Medical University, Taipei, Taiwan

**Keywords:** Acute stroke, Hyaluronic acid, Intracerebral hemorrhage, Ischemic stroke

## Abstract

**Background:**

Activation of hyaluronic acid (HA) and associated enzyme synthesis has been demonstrated in experimental stroke animal models. Our study aimed to investigate the plasma levels of HA in acute stroke patients and the associations between HA levels and functional outcome.

**Methods:**

This was a multicenter case–control study. Acute stroke patients and age- and sex-matched non-stroke controls were recruited. Plasma levels of HA in acute stroke patients were determined at <48 hours and at 48 to 72 hours after stroke onset by standard ELISA. Favorable functional outcome was defined as modified Rankin scale ≤2 at 3 months after stroke.

**Results:**

The study included 206 acute stroke patients, including 43 who had intracerebral hemorrhage and 163 who had ischemic stroke, and 159 controls. The plasma levels of HA in the acute stroke patients were significantly higher than those in the controls (219.7 ± 203.4 ng/ml for <48 hours and 343.1 ± 710.3 ng/ml for 48 to 72 hours versus 170.4 ± 127.9 ng/ml in the controls; both *P* < 0.05). For intracerebral hemorrhage patients, HA ≤500 ng/ml (<48 hours) was an independent favorable outcome predictor (*P* = 0.016). For ischemic stroke patients, an inverted U-shaped association between plasma HA (48 to 72 hours) and outcome was noted, indicating that ischemic stroke patients with too high or too low plasma HA levels tended to have an unfavorable outcome.

**Conclusion:**

HA plasma level was elevated in patients with acute stroke, and can predict 3-month functional outcome, particularly for patients with intracerebral hemorrhage.

## Background

Stroke is the third major cause of mortality and the leading cause of permanent disability worldwide [1,2]. Theoretically, the occurrence of acute stroke would rapidly cause cell necrosis in the affected core region [3-6]. In the region surrounding the core of ischemic or hemorrhagic injures, a series of delayed cellular signaling related to cell apoptosis would then induce more cell death and clinical deterioration [3,7,8]. In the chronic phase, cell repair and regeneration would determine the ultimate extent of brain damage and recovery [9,10]. Among various pathophysiologic mechanisms, inflammatory reactions intrinsic to the central nervous system as well as circulating blood cells are the key elements throughout the different phases after stroke [11,12]. Previous studies have suggested that various inflammatory cytokines(such as tumor necrosis factor-α, interleukin-β, and so forth) modulate tissue injury in an experimental stroke model and have potential as important biomarkers in clinical stroke patients, though their roles in post-stroke outcome are not completely understood [13-17].

Hyaluronic acid (HA) is a ubiquitous glycosaminoglycan composed of repeating disaccharide D-glucuronic acid and N-acetyl-D-glucosamine [18]. It is produced by membrane-bound enzymes called hyaluronan synthases that form D-glucuronic acid and N-acetyl-D-glucosamine disaccharides into a polymer [19]. In the other direction, HA is degraded by hyaluronidase to form oligosaccharides and (very) low molecular weight HA [20]. HA is distributed widely throughout connective, epithelial, and neural tissues [21]. HA has various physiological functions, from acting as the major component of extracellular matrix, cell migration and proliferation, and wound repair, to endogenous activation of immunity [21-24]. Recently, there has been more evidence suggesting that HA degradation products interact directly with toll-like receptors (TLRs) and activate downstream inflammatory signals [25-27].

Importantly, one study showed that hyaluronidases were activated and resulted in the generation of HA fragments in both experimental stroke animal models and human stroke brain tissues [28]. The same study also showed that serum levels of HA in acute ischemic stroke (IS) patients were increased within 1 week after stroke but not associated with stroke severity, though the sample size was small. Moreover, no previous study has focused on the levels of HA in patients with acute intracerebral hemorrhage (ICH). In the present study, we measured the plasma levels of HA in acute stroke patients at two different time points, less than 48 hours and 48 to 72 hours after stroke onset, and aimed to identify the associations between HA levels andfunctional outcome.

## Materials and methods

### Study population

This was a multicenter study involving two medical centers (National Taiwan University Hospital and Tri-Service General Hospital) and three community hospitals (National Taiwan University Hospital Yun-Lin Branch, Taipei Medical University Hospital and Taipei Medical University Shuang Ho Hospital). Patients who had acute stroke and were admitted within 24 hours after stroke onset and who initially had blood drawn within 48 hours after stroke onset were recruited. The diagnosis of stroke was confirmed and characterized by head magnetic resonance imaging (diffusion-weighted imaging) or repeated computed tomographyscanning (if the first scan did not clearly demonstrate the infarct region). Patients with IS or ICH were included. IS was further classified into five major subtypes according to the Trial of ORG 10172 in Acute Stroke Treatment (TOAST) criteria: large artery atherosclerosis, small vessel occlusion, cardioembolism, specific etiology, and undetermined etiology [29]. Patients with a known active infection, cancer, autoimmune disorder, or current steroid treatment at the time of blood sampling were excluded. Age- and sex-matched control subjects free of cardiovascular events within the previous 12 months were included for comparison. This study was approved by the ethics committees of the participating hospitals including National Taiwan University Hospital Research Ethics Committee, Taipei Medical University Joint Institutional Review Board and Institutional Review Board of Tri-Service General Hospital, National Defense Medical Center and all patients gave their written informed consent.

### Clinical protocol

For the acute stroke patients, blood samples were drawn at two time points: within 48 hours and 48 to 72 hours after the onset of stroke. A single sample of blood was taken from each control subject. In our study protocol, blood sampling was performed with at least 18 hours or 1 working day between the time points for the < 48 hours and 48 to 72 hours sample. A detailed history of clinical presentation, vascular risk factors and co-morbidity was obtained from each patient. Medications for diabetes mellitus, hypertension and hyperlipidemia were reviewed and recorded. Body mass index was calculated as weight divided by the square of height. Stroke severity at admission was assessed by the National Institutes of Health Stroke Scale (NIHSS). Mortality and functional outcome 3 months after stroke onset were determined. Favorable outcome was defined as a modified Rankin Scale score of ≤2. Complete blood cell counts and biochemistry were performed at the time of admission.

### Human plasma collection and measurements

Ten-milliliter samples of blood drawn from the controls and stroke patients were decanted into EDTA tubes, then centrifuged at 300 g for 15 minutes, aliquoted into 1.5-ml tubes and stored at -80°C until used. The plasma levels of HA were determined using a commercially available ELISA kit (Hyaluronan Enzyme-Linked Immunosorbent Assay Kit, Echelon Biosciences Inc., Salt Lake City, UT, USA) and no cross reaction to other protein was mentioned according to the manufacturer’s protocol. Measurements were performed in duplicate and the results were averaged. Samples with obvious hemolysis which was visually detected by observing a pink to red tinge inside were not used for measurements.

### Statistical analysis

Statistical analysis was performed using R 2.14.1 software (R Foundation for Statistical Computing, Vienna, Austria). A two-sided *P* value ≤0.05 was considered statistically significant. The distributional properties of continuous variables were expressed as the mean ± standard deviation, whereas categorical variables were represented as frequency and percentage. Forunivariate analysis, the differences in the clinical and biochemical parameters between good and poor outcomes were analyzed using the chi-square test, Fisher’s exact test, the two-sample *t* test, one-way analysis of variance, the Wilcoxon rank-sum test, the Kruskal-Wallis test, or the log-rank test as appropriate. Next, multivariate analysis was conducted using fitting logistic regression models, employing the stepwise variable selection method to estimate the prognostic effects. Generalized additive models (GAM) were applied to detect nonlinear effects of continuous covariates or to discretize them. Importantly, patients with incomplete biochemistry data (n = 3) or missing HA data at either time point (n = 2) were excluded for GAM assay.

## Results

### Plasma levels of hyaluronic acid in acute stroke patients

A total of 206 acute stroke patients (ICH 20.7%) were recruited. The basic characteristics of the study participants are shown in Table 1. Compared with the controls, acute stroke patients had higher HA plasma levels, especially at 48 to 72 hours after stroke onset (219.7 ± 203.4 for <48 hours and 343.1 ± 710.3 for 48 to 72 hours versus 170.4 ± 127.9 ng/ml in controls, *P* = 0.038 and <0.001, respectively). In consideration of stroke subtypes, IS patients were significantly older and had a higher percentage of diabetes mellitus, hypertension and medical treatment for diabetes mellitus than ICH patients. However, both ICH and IS patients had significantly higher HA plasma levels at 48 to 72 hours than at <48 hours post-stroke (all *P* < 0.0001), with the higher levels being more pronounced for ICH patients (*P* = 0.028 compared to IS at 48 to 72 hours).

**Table 1:** Basic demographics of stroke patients and controls

	**Controls (n = 159)**	**Stroke patients (n = 207)**	**Stroke subtypes**	
			**ICH (n = 43)**	**IS (n = 163)**
Age (years)	62.7 ± 7.5	64.3 ± 13.8	60.1 ± 14.7	65.4 ± 13.5*
Male	90 (56.6)	139 (67.1)	28 (65.1)	111 (68.1)
Body mass index (kg/m^2^)	25.7 ± 4.1	25.4 ± 3.8	24.0 ± 3.7	25.7 ± 3.8
Diabetes mellitus	77 (48.4)	77 (37.2)	10 (23.3)	67 (41.1)*
Hypertension	95 (59.7)	158 (76.3)	39 (90.7)	119 (73.0)*
Hyperlipidemia	40 (25.2)	62 (30.0)	9 (20.9)	53 (32.5)
Medication for:				
Diabetes mellitus	NA	58 (28.0)	5 (11.6)	53 (32.5)*
Hypertension	NA	113 (54.6)	22 (51.1)	91 (55.8)
Hyperlipidemia	NA	28 (13.5)	3 (7.0)	25 (15.3)
HA <48 hours (ng/ml)			252.6 ± 188.3	211.0 ± 206.9
HA 48 to 72 hours (ng/ml)			554.0 ± 1127.7	287.1 ± 540.7*

Values are shown as number (percentage) or mean ± standard deviation. HA, hyaluronic acid; ICH, intracerebral hemorrhage; IS, ischemic stroke; NA, not available. *P < 0.05 versus ICH.

### Plasma levels of hyaluronic acid (<48 hours) predicts outcome in intracerebral hemorrhage patients

In ICH patients, univariate analysis showed that patients with favorable outcome (n = 23, 53.5%) were younger, had higher body mass index values, and lower NIHSS scores than those with unfavorable outcome (Table 2). HA plasma levels tended to be lower in patients with favorable outcome at <48 hours and 48 to 72 hours (*P* = 0.06 and 0.08, respectively).

**Table 2:** Comparison by functional outcome for patients with intracerebral hemorrhage and ischemic stroke

	**Intracerebral hemorrhage**			**Ischemic stroke**		
	mRS ≤2* (n = 23)	**mRS > 2 (n = 20)**	***P***	mRS ≤2* (n = 82)	**mRS > 2 (n = 81)**	***P***
Age (years)	54.2 ± 12.9	66.8 ± 13.9	**0.004**	62.6 ± 14.0	68.3 ± 12.4	**0.007**
Male	15 (65.2)	13 (65)	1.000	64 (78.0)	47 (58.0)	**0.007**
Body mass index (kg/m^2^)	25.1 ± 3.1	22.6 ± 3.8	**0.023**	25.9 ± 3.8	25.5 ± 3.7	0.528
Diabetes mellitus	8 (34.7)	2 (10.0)	0.076	30 (36.6)	37 (45.7)	0.267
Hypertension	21 (91.3)	18 (90.0)	1.000	53 (64.6)	66 (81.5)	**0.021**
Hyperlipidemia	3 (13.0)	6 (30.0)	0.263	25 (30.5)	28 (34.6)	0.618
Atril fibrillation	1 (4.3)	1 (5.0)	1.000	17 (20.7)	25 (30.9)	0.155
History of stroke	3 (13.0)	4 (20.0)	0.687	10 (12.2)	26 (32.1)	**0.002**
WBC (10^3^/μl)	10.2 ± 4.4	10.3 ± 2.8	0.860	8.3 ± 2.8	9.3 ± 4.0	**0.050**
Hemoglobin	14.9 ± 1.3	13.0 ± 4.4	0.057	14.6 ± 3.7	13.9 ± 1.9	0.173
Glucose (mg/dl)	137.3 ± 59.2	113.5 ± 32.3	0.117	145.5 ± 61.9	148.8 ± 55.8	0.723
Creatinine (mg/dl)	1.2 ± 0.7	1.1 ± 0.4	0.492	1.2 ± 1.0	1.3 ± 0.9	0.356
NIHSS	9.7 ± 5.5	17.6 ± 7.6	**<0.001**	5.1 ± 5.8	13.1 ± 7.4	**<0.001**
Systolic blood pressure (mmHg)	184.1 ± 44.5	190.1 ± 38.2	0.640	157.9 ± 31.8	160.8 ± 32.7	0.565
HA < 48 hours (ng/ml)	202.7 ± 134.1	310.1 ± 225.9	0.061	195.9 ± 164.0	226.0 ± 242.6	0.356
HA 48 to 72 hours (ng/ml)	271.1 ± 226.8	879.6 ± 1594.7	0.077	206.1 ± 304.2	379.2 ± 698.0	0.053

Values are shown as number (percentage) or mean ± standard deviation. *P* values in bold indicate statistical significance. HA, hyaluroninc acid; mRS, modified Rankin scale; NIHSS, National Institute of Health Stroke Scale; WBC, white blood cell. *Favorable outcome is mRS ≤2 at 3 months after stroke; unfavorable outcome is mRS > 2 at 3 months after stroke.

**Figure 1:**
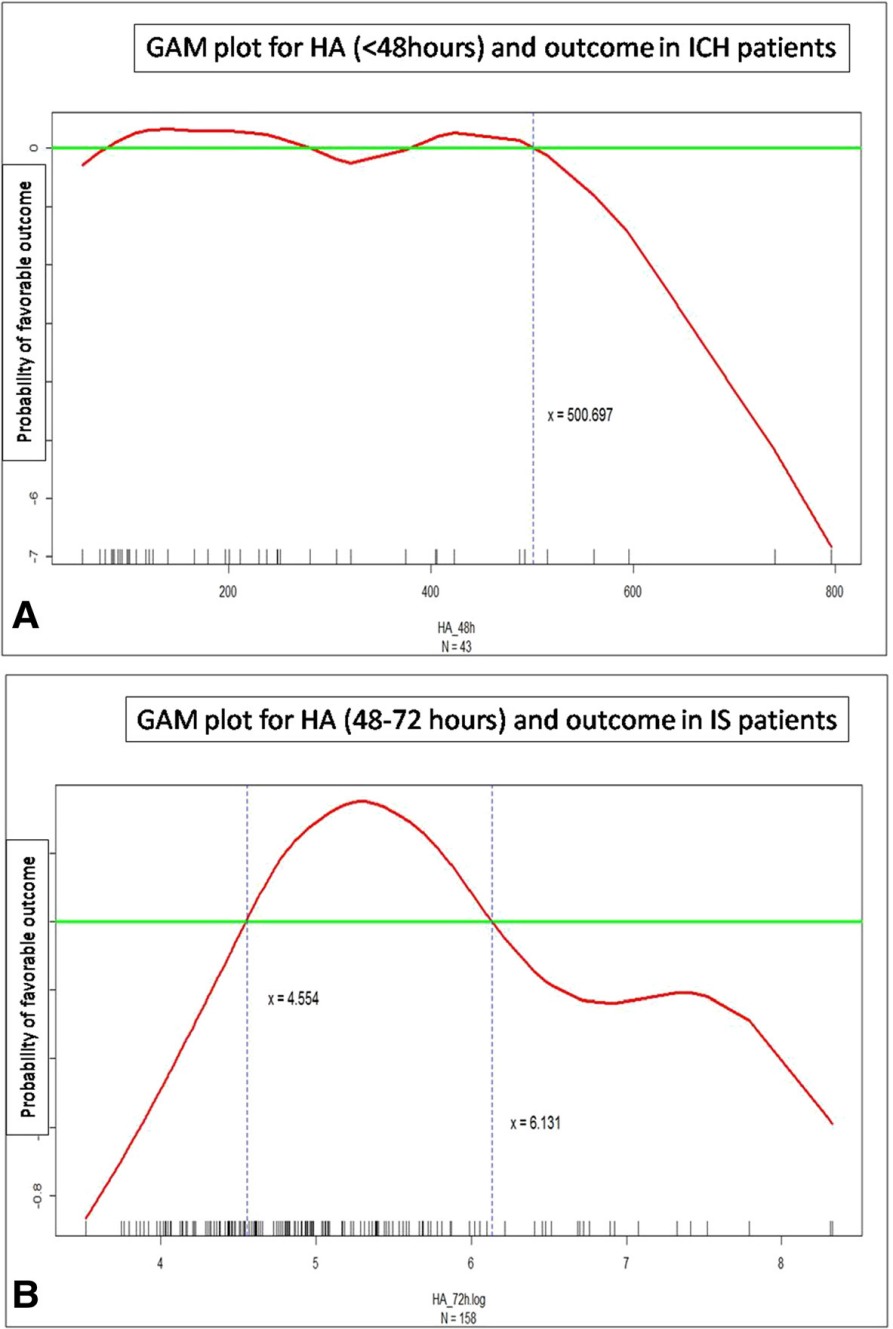
**Association between plasma levels of hyaluronic acid and post stroke outcome. (A)** For intracerebral hemorrhage (ICH) patients, generalized additive models (GAM) identified a cutoff value of hyaluronic acid (HA) ≤500 ng/ml at <48 hours after stroke onset in reference to a favorable outcome after adjustment for National Institutes of Health Stroke Scale (NIHSS), diabetes mellitus and age. **(B)** Incontrast, there was an inverted U-shaped relationship between log transformed plasma levels of HA at the 48 to 72 hours time point and outcome in ischemic stroke (IS) patients after adjustment for NIHSS, initial glucose levels, sex, history of dyslipidemia and history of stroke.

Importantly, GAM identified a cutoff value of HA ≤ 500 ng/ml at <48 hours after stroke onset in reference to a favorable outcome after adjustment for NIHSS score, diabetes mellitus and age (Figure 1A). Multivariate analysis showed that plasma HA ≤500 ng/ml at <48 hours can significantly predict a favorable outcome at 3 months after ICH (*P* = 0.016) when adjusted for age, body mass index, and NIHSS score.

### Inverted U-shaped relationship between plasma hyaluronic acid (48 to 72 hours) and outcome in ischemic stroke patients

There were 91 (55.5%) IS patients with anterior circulation infarction; the others (n = 72) had posterior circulation infarction. Univariate analysis showed that variables correlated with favorable outcome included younger age, male gender, lower NIHSS score and white blood cell count at admission, no history of hypertension and no history of stroke (Table 2). Similarly to ICH, HA plasma levels tended to be lower in IS patients with a favorable outcome at 48 to 72 hours (*P* = 0.053). With regard to IS subtypes based on the TOAST classification, patients with cardioembolism had significantly higher NIHSS scores and higher HA levels (48 to 72 hours) than patients with all the other subtypes (Table 3).

**Table 3:** Basic demographics and hyaluronic acidlevels in ischemic stroke patients with different subtypes based on the TOAST classification

	**Large-artery atherosclerosis (n = 47)**	**Small vessel occlusion (n = 44)**	**Cardio-embolism (n = 30)**	**Others (n = 42)**
Age (years)	64.0 ± 12.8	65.0 ± 13.7	68.8 ± 11.9	64.9 ± 15.2
Male	37 (78.7)	31 (74.5)	18 (60.0)	25 (59.5)
Body mass index (kg/m^2^)	25.4 ± 3.2	26.4 ± 3.9	24.9 ± 3.0	25.9 ± 4.2
Diabetes mellitus	23 (48.9)	16 (36.4)	11 (36.7)	17 (40.5)
Hypertension	33(70.2)	29 (65.9)	23 (76.7)	34 (81.0)
Hyperlipidemia	16 (34.0)	16 (36.4)	5 (16.7)	16 (38.1)
Atril fibrillation	0 (0)	0 (0)	26 (74.1)**	16 (38.1)**
History of stroke	14(30.0)	7(15.9)	9 (30.0)	6 (14.3)
NIHSS	10.9 ± 8.4**	4.7 ± 4.6	14.9 ± 6.2**	7.6 ± 7.6
mRS ≤2	17 (36.2)	29 (65.9)**	9 (30.0)	27 (64.3)**
HA <48 hours (ng/ml)	194.7 ± 186.4	213.4 ± 258.3	258.6 ± 191.5	192.2 ± 178.3
HA (ng/ml) 48 to 72 hours (ng/ml)	218.8 ± 270.41	189.5 ± 260.8	508.4 ± 879.8^*^	316.2 ± 644.7

Values are shown as number (percentage) or mean ± standard deviation. HA, hyaluronic acid; mRS, modified Rankin scale; NIHSS, National Institute of Health Stroke Scale; Others, specific etiology, and undetermined etiology in TOAST classification; TOAST, Trial of ORG 10172 in Acute Stroke Treatment.

The values of all pairs of columns were compared to each other.*P < 0.05, **P < 0.01.

Although multivariate analysis did not show an independent effect of HA plasma levels at either <48 hours or 48 to 72 hours in predicting outcome, GAM demonstrated an inverted U-shaped relationship between log transformed HA plasma levels at 48 to 72 hours and outcome, which indicates that patients with values of HA(_log transformed_) between 4.55 and 6.12 ng/ml tend to have a higher probability of favorable outcome than others after adjustment for independent clinical parameters including NIHSS score, initial glucose levels, sex, history of dyslipidemia and history of stroke (*P* = 0.095) (Figure 1B).

## Discussion

TLRs and associated downstream signaling in the central nervous system has been shown to be upregulated in response to acute ischemic or hemorrhagic strokes [30-34]. Importantly, activation of TLR signaling requires ligands. Possible candidates of endogenous TLR ligands include high mobility group box 1 (HMGB1), HA and heat shockproteins, and so forth [35]. Our recent work showed that plasma levels of HMGB1 increased after the onset of IS, and inhibition of HMGB1 activity via administration of a recombinant-soluble form of receptor for advanced glycation end-products significantly improved outcome in a mouse stroke model [36]. However, data from our IS patients did not show a significant association between the plasma levels of HMGB1 and 3-month functional outcome [36]. One possibility may be the relatively small sample size in that study. In addition, we did not measure plasma levels of HMGB1 in ICH patients. Further studies to compare the correlation between plasma HMGB1 and HA at the same time points would be interesting.

The current study revealed several important findings. First, the levels of plasma HA were significantly higher in acute stroke patients than in controls and the levels increased progressively after stroke. Second, lower levels of plasma HA at the time point of <48 hours after stroke with a cutoff level of HA ≤500 ng/ml tended to indicate a better prognosis in ICH patients. Third, there is an inverted U-shaped relationship between plasma HA level at 48 to 72 hours after stroke onset and post-stroke outcome in IS patients.

Previous studies have shown increased levels of circulating HA in patients with various diseases, such as septic shock, thermal injury, rheumatoid arthritis and vasculitis [37-41]. Most studies showed that HA levels are correlated with an increase in disease severity. These data support the concept that a systemic inflammatory process may enhance the production of HA and trigger the associated immune responses. Elevated plasma HA has been found to be a marker for the extent of collateral formation in patients with coronary artery disease [42]. Furthermore, in our study, IS patients had a higher percentage of medical treatment for diabetes mellitus but not for hypertension and hyperlipidemia than ICH patients. Whether medication for cardiovascular diseases would affect the levels of HA remains uncertain and deserves further investigation.

For patients with acute stroke, only one previous study(that included 54 acute IS patients) showed that serum HA levels increased significantly, with the highest levels found at 7 days after stroke [28]. However, there was no obvious association between hyaluronidase activity and clinical parameters, including post-stroke recovery. Together with our findings, these results may highlight the complicated role of HA-mediated inflammatory responses in the pathogenesis of acute ischemic brain injury. Looking back at previous relevant studies, acute immune responses were initially recognized as key detrimental elements for post-ischemic cell death after stroke. However, anti-inflammatory therapies did not improve outcome in clinical settings [43]. Therefore, the finding in our IS patients of an inverted U-shaped relationship between plasma HA and outcome may exactly reflect the dilemma that delayed inflammation seems to be a double-edged sword in post-IS damage and recovery. With respect to IS subtypes, patients with cardioembolism had significantly higher levels of HA than patients with the other IS subtypes. They also had higher NIHSS scores than patients with other IS subtypes. Thus, the differences in HA levels among different IS subtypes may be related to the severity of acute stroke rather than the IS subtype.

Compared to IS, research on the role of inflammation after ICH has been more limited. Available evidence from preclinical and clinical studies suggest that inflammatory mechanisms are also involved in secondary brain injury and recovery in ICH, though the importance of their contribution to post-stroke outcome is still uncertain [17]. In our study, the linear association between plasma levels of HA (<48 hours) and outcome in ICH patients leads to the detrimental implication of the immune response in the pathogenesis of post-ICH brain damage.

There are several study limitations. First, acute stroke could trigger primary inflammation in the affected brain region and secondary systemic inflammation. Thus, it is unclear whether our measured plasma HA levels were influenced predominantly by the central nervous system or systemic systems. Second, the functions of HA could be diverse, based on the molecular weights of HA and the activities of hyaluronidase [21]. The results from our analytic method (ELISA) showed the total amount of plasma HA rather than the HA within a specific range of molecular weights. Whether this factor can have a large effect on the interpretation of our results remains to be determined by further investigation. However, so far there is no clinically useful way to quantify a specific range of plasma HA levels in human subjects. The last limitation is that the sample size of ICH patients was relatively small, though the results were statistically significant. Further studies with larger sample sizes are necessary to strengthen our findings.

In conclusion, our study provided evidence of activation of HA synthesis in acute stroke and identified the diverse patterns of association between plasma HA levels and outcome in patients with IS and ICH. Furthermore, levels of HA after stroke can help predict functional outcome, especially in ICH patients.

## Abbreviations

ELISA: enzyme-linked immunosorbent assay; GAM: generalized additive models; HA: hyaluronic acid; HMGB1: High mobility group box 1; ICH: intracerebral hemorrhage; IS: ischemic stroke; NIHSS: National Institutes of Health Stroke Scale; TLR: toll-like receptor; TOAST: Trial of ORG 10172 in Acute Stroke Treatment.

## Competing interests

The authors declare that they have no competing interests.

## Authors’ contributions

SCT, SJY, LKT, CJH, LML and GSP collected patient samples. SCT, WSY, HYC and JSJ performed the data analysis. SCT and JSJ wrote the manuscript. SJY, LKT and JSJ revised the manuscript. All authors read and approved the final manuscript.
